# Serum S100β and neuron-specific enolase correlate with obesity parameters in Mexican children

**DOI:** 10.1038/s41366-025-01942-y

**Published:** 2025-11-24

**Authors:** Gabriela Hurtado-Alvarado, Rebeca Mendez-Hernandez, Karol Iliana Avila-Soto, Alberto Salazar-Juárez, Mónica Espinoza-Rojo, Carolina Escobar, Miguel Vázquez-Moreno, Miguel Cruz

**Affiliations:** 1 Department of Anatomy, Faculty of Medicine, National Autonomous University of Mexico, Copilco Universidad, Coyoacán, Mexico City, Mexico; 2 Monell Chemical Senses Center, Philadelphia, PA USA; 3 Medical Research Unit in Biochemistry, Specialties Hospital, National Medical Center Siglo XXI, Mexican Social Security Institute, Doctores, Cuauhtémoc, Mexico City, Mexico; 4 Branch Clinical Research, Laboratory of Molecular Neurobiology and Neurochemistry of Addiction, National Institute of Psychiatry “Ramón de la Fuente”, Huipulco, Tlalpan, Mexico City, Mexico; 5 Laboratory of Genomic and Molecular Biology, Autonomous University of Guerrero, Chilpancingo de los Bravo, Guerrero, Mexico

**Keywords:** Obesity, Pre-diabetes

## Abstract

**Background:**

Circulating S100 β and neuron-specific enolase (NSE) have been used to explore brain damage in adults with obesity. Nonetheless, the subtle increase of these molecules can be found in non-pathological conditions in healthy subjects, indicating possible disturbances in brain function.

**Objective:**

We aimed to compare serum levels of S100β and NSE between children with and without obesity.

**Subjects and methods:**

We analyzed circulating S100β and NSE and performed correlations with anthropometry and biochemical parameters from 80 children between 6 and 11 years old, divided into two groups: children with obesity (Body mass index ≥97th percentile) and children with normal body weight (between the 5th and 85th percentile).

**Results:**

Our results show that children with obesity have approximately 50% more circulating levels of S100β and NSE. Furthermore, we found a positive correlation between S100β and circulating resistin and a positive correlation between NSE and Body mass index, waist circumference, and waist-to-hip ratio. Conversely, NSE and adiponectin showed a negative correlation.

**Conclusion:**

S100β and NSE levels in blood were associated with indicators of metabolic impairment. Future studies are needed to determine if the increase of S100β and NSE in children with obesity is related to cognitive function.

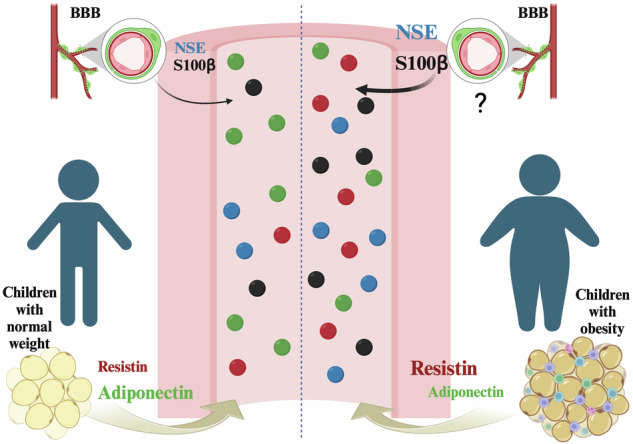

## Introduction

S100 calcium-binding protein (S100)-β, neuron-specific enolase (NSE), and brain-derived neurotrophic factor (BNDF) are produced and secreted by neural cells [1]. The concentrations of these molecules in circulation have consistently been found elevated in pathologies such as traumatic brain injury and conditions associated with cognitive impairment [1, 2]. Beyond clinical conditions, S100β and NSE are also elevated in non-pathological conditions such as internet addiction [3] or acute sleep deprivation [4], suggesting that high circulating levels of S100β and NSE might derive from increased brain leakage due to BBB dysfunction or higher production by external sources (e.g., adipose tissue [5]) rather than as a direct consequence of neuronal damage as in traumatic brain injury [6].

In adults with obesity, circulating S100β protein and NSE positively correlate with waist circumference and body mass index (BMI) [7]. Moreover, a study found that reduced gray matter density, measured by magnetic resonance imaging (MRI), correlated with NSE levels in circulation in subjects with overweight and obesity (aged 20–41) [8], even when NSE serum levels were in the non-pathological range (1.83–10 ng/mL) [9]. These data suggest a link between BBB disruption, obesity, and brain function that warrants further investigation.

An essential link between BBB dysfunction and obesity is the low-grade inflammatory state [10]. Notably, in children, obesity is associated with inflammation and decreased adiponectin levels, an anti-inflammatory and neuroprotective hormone [11]. In addition, children with obesity exhibit cognitive impairments, such as decreased attention, executive functioning, and motor skills [12–17], and higher levels of circulating molecules related to the risk of developing neurodegenerative diseases [18]. These data highlight the importance of understanding the relationship between obesity and BBB dysfunction in younger populations.

In the present study, we aimed to assess circulating concentrations of NSE, S100β, and BDNF in children with obesity. In addition to higher levels of circulating S100β protein and NSE in children with obesity, we identify high levels of resistin and lower levels of adiponectin, suggesting a proinflammatory status in these children.

## Materials and methods

### Participants

A total of 1559 children were recruited for project R-2012-785-071, which aims to characterize the biochemical and anthropometric characteristics of Mexican Children and generate a metabolic certificate and medical intervention.

For this descriptive and comparative cross-sectional study, a subset of those individuals was selected by convenience sampling by age (between 6 and 11) and sex (females 50%). The sample size calculation for comparing two means used reference estimates from previous studies [19]. Despite the minimal necessary sample size of at least 23 in each group, we selected 40 subjects per group. Forty children with normal weight (BMI between the 5th and 85th percentile BMI percentile 1) and 40 children with obesity (BMI of ≥ 97th percentile: BMI percentile 3) based on the WHO criteria of percentiles for overweight and obesity for children between 5 and 19 years old (available at https://www.who.int/tools/growth-reference-data-for-5to19-years/application-tools) were included in this study. Blood was collected from each participant by trained hospital personnel in Vacutainer™ serum separator tubes and stored at −80 °C until use. Inadequate or insufficient samples (e.g., samples with hemolysis) were eliminated from the study. Recruited children received a clinical examination conducted by qualified personnel, which included a family history questionnaire and anthropometric measurements. Children with chronic or acute infectious diseases and those undergoing weight-reduction programs (with or without pharmacological treatment) were excluded from the study.

### Biochemical measurements

Serum aliquots from the same individuals were used to measure biochemical parameters and biomarkers of BBB dysfunction. Serum concentrations of hormones (insulin, leptin, adiponectin, resistin) and metabolites (glucose, total cholesterol, HDL cholesterol, LDL cholesterol, triglycerides) were measured from blood samples processed at the Medical Research Unit in Biochemistry using the equipment iLab Aries and the QuantILab Clinical Chemistry reagents (required serum volume: 50 μL). Serum levels of NSE were measured using the MILLIPLEX® products that are based on the Luminex® xMAP® technology and NSE (MILLIPLEX® Human Cancer Metastasis Biomarker Magnetic Bead Panel 96-Well Plate Assay HCMBMAG-22K). Serum samples were diluted and processed with the standard, high, and low controls according to the protocol provided by the manufacturer.

S100β was quantified using an Enzyme-Linked Immunosorbent Assay (Human S100β ELISA 96-Well Plate Cat. # EZHS100B-33K). ELISA was performed as recommended by the manufacturer, and absorbance was read at 450 nm using a BioTek Epoch Microplate Spectrophotometer. BDNF was measured using an Enzyme-Linked Immunosorbent Assay (Human BDNF ELISA 96-Well Plate Cat. # EH42RB). The ELISA was performed as recommended by the manufacturer, and absorbance was measured at 450 nm using a BioTek Epoch Microplate Spectrophotometer.

### Statistics

Data were tested for normal distribution using the Kolmogorov–Smirnov test. The variables that showed normal distribution were analyzed using the Student’s *t* test, while those that did not show a normal distribution were analyzed via Mann–Whitney *U* tests. The Levene test was used to assess variance homogeneity. We calculated the Pearson or Spearman correlation coefficient between variables depending on the data distribution. Furthermore, we performed a main-effects multivariate linear regression analysis (Least Squares) to identify the anthropometric and biochemical variables that predicted NSE, S100β, and BNDF levels. Correlations were not performed for individuals with missing data for any given variable. Statistical significance was set at *p* < 0.05. GraphPad Prism version 9 was used for data processing and analysis. All normally distributed data are presented as the mean ± standard error of the mean (SEM), and non-normal data are presented as the median ± interquartile range.

## Results

### Descriptive characteristics of the population

Anthropometric and biochemical characteristics of the 80 Mexican children (boys 50%, girls 50%) are indicated in Table 1. Children in the BMI percentile 3 weighed roughly twice as much as children in the BMI percentile 1 (Table 1). Height, BMI, waist-hip circumference, and blood pressure values were also higher in the group of children with obesity than in the group of BMI percentile 1 (Table 1).

**Table 1:** Descriptive characteristics of the population.

Variable	Percentile 1 Normal weight children (*n* = 40)	Percentile 3 Children with obesity (*n* = 40)	*p* value
Females (%)	50	50	
Anthropometry			
Age (years)^a^	8 ± 1.43	8 ± 1.43	>0.9999
Weight (kg)^a^	24.35 ± 7.90	44.55 ± 20.55	<0.0001*
Height (m)^b^	1.23 ± 0.10	1.36 ± 0.11	<0.0001*
Body mass index (kg/m^2^)^b^	15.53 ± 1.18	24.86 ± 3.40	<0.0001*
Waist circumference (cm)^b^	54.50 ± 4.33	79.6 ± 8.35	<0.0001*
Hip circumference (cm)^b^	65.10 ± 5.91	87.2 ± 9.04	<0.0001*
Waist-Hip Ratio^b^	0.84 ± 0.04	0.91 ± 0.04	<0.0001*
Systolic blood pressure (mmHg)^b^	92.00 ± 9.09	102.00 ± 12.10	0.0001*
Diastolic blood pressure (mmHg)^a^	60.00 ± 40.75	70.00 ± 14.25	0.0033*
Biochemical markers			
Glucose (mmol/L)^b^	4.40 ± 0.58	4.64 ± 10.8	0.0830
Total cholesterol (mmol/L)^b^	4.19 ± 0.86	4.37 ± 34.9	0.3813
LDL cholesterol (mmol/L)^b^	2.71 ± 0.76	2.98 ± 26.3	0.0960
HDL cholesterol (mmol/L)^b^	1.48 ± 0.32	1.13 ± 11.4	<0.0001*
Triglycerides (mmol/L)^a^	0.79 ± 0.29	1.54 ± 84	<0.0001*
Insulin (μUI/mL)^a^	5.72 ± 1.40	11.0 ± 3.17	0.0024*
HOMA-IR Index^a^	1.15 ± 0.48	2.34 ± 1.13	0.0026*

Data are expressed as mean ± standard deviation depending on data normality %. All normally distributed data are presented as the mean ± standard error of the mean (SEM), and non-normal data are presented as the median ± interquartile range. Differences between groups were analyzed using Student’s *t* test and Mann–Whitney *U* test. Data were assessed for normality and differences in means were analyzed using Student’s *t* test.

*LDL* low-density lipoprotein, *HDL* high-density lipoprotein, *HOMA-IR* homeostatic model assessment insulin resistance.

*p value < 0.05.

^a^Normal distribution—parametric statistical methods.

^b^Free distribution—nonparametric statistical methods.

Bold/italicized values highlight the significant differences.

### Biochemical markers

Levels of HDL cholesterol, triglycerides, insulin, and the HOMA Index were higher in the group of children in the BMI percentile 3 group than in the BMI percentile 1 group. Glucose concentrations, total cholesterol, and LDL cholesterol did not differ significantly between groups (Table 1).

**Fig. 1:**
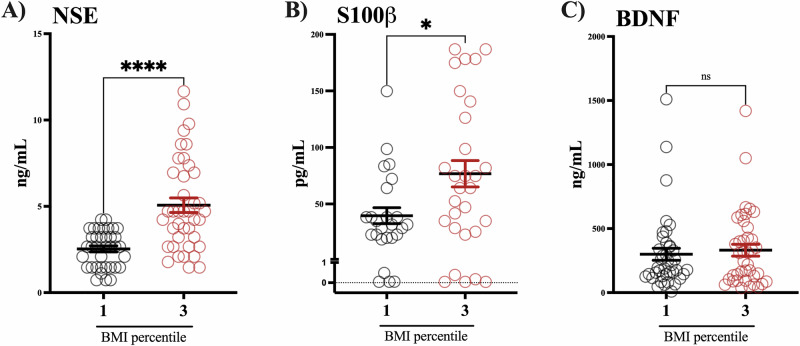
Children with obesity exhibit higher levels of NSE and S100β. The graph shows S100β (**A**), NSE (**B**), and BNDF (**C**) serum levels in children with normal weight (*n* = 40) and obesity (*n* = 40). Mann–Whitney test. mean ± SEM. *p > 0.05, ****p > 0.0001.

**Fig. 2:**
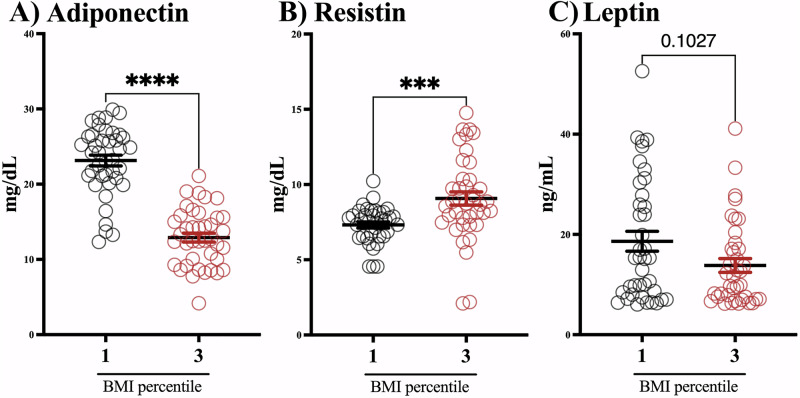
Adipokine levels are altered in children with obesity. The graph shows adiponectin (**A**), resistin (**B**), and leptin (**C**) serum levels in children with normal weight (BMI percentile 1) (*n* = 40) and obesity (BMI percentile 3) (*n* = 40). Mann–Whitney test. Data are expressed as the mean ± SEM. ***p > 0.001, ****p > 0.0001.

Children in the BMI percentile 3 group had roughly double the concentration of NSE and S100β than the BMI percentile 1 group (Fig. 1A, B); meanwhile, no differences were found in the serum levels of BDNF (Fig. 1C). We evaluated the circulating levels of adipokines, and we found that adiponectin levels were lower in the group of children in the BMI percentile 3 compared to the BMI percentile 1 group (Fig. 2A). Conversely, children in the BMI percentile 3 had higher resistin levels than those with normal weight (Fig. 2B). We did not find differences in leptin levels between groups (Fig. 2C).

**Fig. 3:**
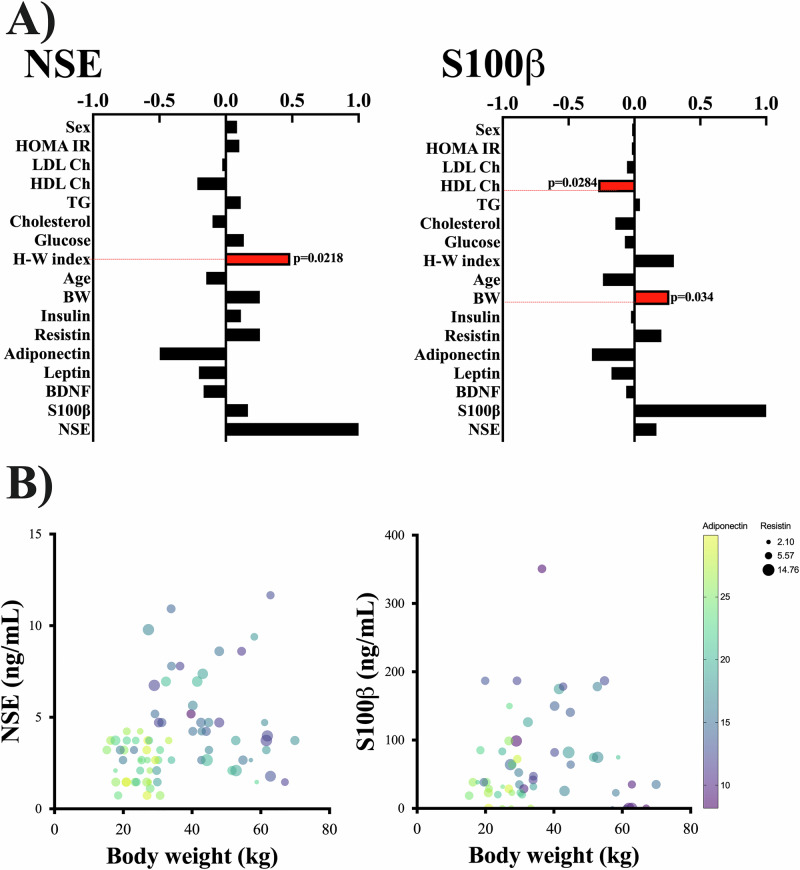
NSE and S100β correlate with anthropometric parameters in children with obesity. The graphs showed the correlations of S100β and NSE with biochemical and anthropometric parameters in 80 children (**A**). Bubble plots represent the association of NSE and S100β with body weight in 80 Mexican children. The levels of adiponectin (color) and resistin (size) are included to illustrate the differences in children depending on the body weight (**B**).

The correlation analysis showed that NSE correlated negatively with adiponectin (*r* = −0.417), waist circumference (*r* = −0.347), and waist-hip ratio (*r* = −0.447), all indicators of metabolic syndrome. Moreover, S100β positively correlated with circulating resistin levels (*r* = 0.352). We found no correlations between BDNF and any of the anthropometric or biochemical variables (Supplementary Table 1). Furthermore, age and sex did not correlate with S100β, NSE, or BNDF. The multivariate linear analysis, which accounts for multiple variables in contrast to the correlations, was an adequate model to predict NSE and S100β (*p* = 0.0002 and *p* = 0.0203, respectively). These two variables were associated with the BMI percentile (Table 2), aligning with the previous results showing clear differences in these two markers between the groups. The main-effects multivariate linear regression analysis (Least Squares) to identify the anthropometric and biochemical variables that predicted NSE, S100β, and BNDF levels showed that NSE correlates with BMI percentile (*p* = 0.0094) and Hip-waist index (0.0218); S100β correlates with body weight (*p* = 0.0034), BMI percentile (*p* = 0.0124) and HDL-Cholesterol (*p* = 0.0284) (Table 2 and Fig. 3). In contrast, the linear model did not predict BDNF with any variables (*p* = 0.7417).

**Table 2:** Results from the multiple linear regression analysis for NSE and S100β.

Independent variable	Dependent variable NSE		Dependent variable S100β (log)	
	*β* ± S.E.	*p* value	*β* ± S.E.	*p* value
Leptin	−0.009 ± 0.025	0.7148	−0.004 ± 0.015	0.7998
Adiponectin	−0.053 ± 0.065	0.4171	0.001 ± 0.037	0.9732
Resistin	−0.03 ± 0.114	0.79	0.013 ± 0.062	0.8369
Insulin	0.473 ± 0.3	0.1208	−0.116 ± 0.217	0.5952
BW	−0.107 ± 0.055	0.0569	−0.096 ± 0.031	0.0034*
Age	0.571 ± 0.372	0.1306	0.412 ± 0.229	0.0795
BMI percentile	4.018 ± 1.495	0.0094*	1.187 ± 0.453	0.0124*
H-W index	13.79 ± 5.85	0.0218*	−2.35 ± 3.34	0.4859
Glucose	0.05 ± 0.036	0.1637	−0.003 ± 0.022	0.8892
Cholesterol	−0.018 ± 0.022	0.4234	0.008 ± 0.014	0.5947
Triglycerides	−0.004 ± 0.007	0.5688	−0.004 ± 0.004	0.3284
HDL Cholesterol	0.022 ± 0.028	0.437	−0.038 ± 0.017	0.0284*
LDL Cholesterol	0.015 ± 0.024	0.5297	−0.017 ± 0.016	0.2762
HOMA-IR	−2.399 ± 1.519	0.12	0.609 ± 1.067	0.5712
Sex	0.013 ± 0.53	0.9809	0.358 ± 0.299	0.2392

*NSE* neuron-specific enolase, *BW* body weight, *BMI* body mass index, *H-W* hip-waist, *LDL* low-density lipoprotein, *HDL* high-density lipoprotein, *HOMA-IR* homeostatic model assessment insulin resistance, *S.E.* standard error.

Bold/italicized values highlight the significant differences.

*p value > 0.05

## Discussion

The increasing prevalence of overweight and obesity, key components of the metabolic syndrome, in the pediatric population poses a high risk of health complications, including the development of Type 2 diabetes [20]. As expected, we determined that our cohort of children with obesity has higher BMI, waist-hip circumference, and blood pressure values compared to the control group. In addition, the group of children with obesity has insulin resistance and higher circulating levels of triglycerides. Our data are similar to other reports in Mexican children with bigger cohorts that, in addition, reported that Mexican children with obesity present glucose intolerance [11, 21].

It has been suggested that obesity and insulin resistance may promote neuroinflammation, promoting brain dysfunction [22]. Moreover, childhood obesity is associated with compromised executive function and significantly reduced mean cortical thickness in prefrontal cortical regions [23]. Another study demonstrated that circulating macrophages that induce neuroinflammation in animal models of obesity [24] are higher in children with obesity, who have lower cognitive performance than the control group [25]. Children with obesity and metabolic disturbances also exhibit a circulatory proteomic profile enriched with proteins related to neurological disorders and BBB dysfunction [26]. In addition, insulin resistance and high levels of triglycerides, both present in children with obesity, alter BBB function [27, 28]. These studies further support the relationship between neuroinflammation-induced BBB disruption, obesity, and cognitive performance, and are consistent with animal studies with genetic or diet-induced obesity [29–32].

Measuring circulating molecules from the brain is a recurrent approach to exploring possible neuronal damage by BBB dysfunction that allows the release of molecules to the bloodstream. S100β, NSE, and BDNF are some of the most extensively studied biomarkers for neuronal damage that have been shown to positively correlate with brain dysfunction, neuroinflammation, neuronal activity, and cognitive dysfunction in several neuropathologies [33]. Nonetheless, the rise of S100β and NSE in serum is also observed in non-pathological conditions such as sleep loss [9].

Here, we show that circulating levels of S100β and NSE are higher in children with obesity. The higher levels of S100β and NSE in the bloodstream may indicate impaired BBB function; however, the range of S100β and NSE in children with obesity remains within the non-pathological range. For instance, NSE levels in children with obesity (5.07 ± 2.66 ng/mL) are approximately 3- or 125-fold time lower than those reported in children with Type 1 diabetes (NSE, 14.6 ng/mL) [34] or those with extreme brain injury (NSE > 250 ng/mL) [35]. In adults with obesity, S100β is approximately 20% higher than in controls [19]. We found an approximately 50% increase in circulating levels of S100β. High levels of S100B correlated with abdominal obesity, serum levels of triglycerides, and insulin resistance in adults [36]. Certainly, S100β is abundant in brain astrocytes; however, recent studies have also identified adipose tissue as a source of protein concentration. Thus, in addition to the possible BBB dysfunction, adipose S100β could rise as an essential signal in obesity that needs to be studied to elucidate if there is a differential proportion in the production of S100β between children and adults. Furthermore, in young people, BMI (9–18 years) is a weaker predictor of relative body fat [37, 38]; therefore, S100β as a marker of high adiposity or BBB dysfunction should be evaluated carefully, particularly in this young population. Instead, the waist-hip ratio indicates cardiovascular disease risk and beta pancreatic function in children and adults [39]. In addition, children with obesity had higher levels of NSE, as reported in adults [8]. NSE is also positively correlated with the waist-hip ratio.

We found that S100β negatively correlated with resistin, a proinflammatory molecule that may play a role in obesity, insulin resistance, type 2 diabetes, inflammation, cancer, and atherosclerosis [40, 41]. The increased release of S100β induces lipolysis, leading to a rise in the triglyceride levels and inducing inflammation in adipose tissue [5]; therefore, we could speculate that S100β contributes to the development of dyslipidemia in children with obesity. The higher NSE levels have been associated not only with the occurrence of vascular events such as stroke and vascular headache but also with an earlier presentation of hypertension [8]. Although we did not find a correlation between NSE and blood pressure indicators, children with obesity had higher blood pressure. In addition, it has been reported that as childhood obesity rates increase worldwide, the prevalence of obesity-related hypertension is also on the rise [42, 43] and might impact brain function. Indeed, obesity and hypertension in adults are considered risk factors for brain dysfunction [44, 45].

We found a decrease in adiponectin levels in children with obesity that negatively correlates with NSE. Previous studies reported that in children with obesity, as adiponectin concentrations decrease, the prevalence of components of the metabolic syndrome, including higher values of waist circumference, systolic blood pressure, diastolic blood pressure, triglycerides, and HDL-Ch, increases [11, 21].

The downregulation of adiponectin production is reported in adult patients with obesity, and it can be upregulated once obesity is reduced [46], which indicates that this adipokine has an essential value as a predictor of adiposity. Adiponectin has also been considered a potential biomarker of cognitive decline in patients with cognitive impairment [47, 48]. A systematic review based on 43 published studies suggests that adiponectin links obesity and cognitive impairment [49]. In addition, a Mendelian randomization, a genetics-based approach in epidemiological research, suggests that lower adiponectin is associated with poor cognitive performance in subjects with obesity [50]. The link between adiponectin and cognitive impairment is sustained by the fact that lower adiponectin levels in subjects with obesity lead to insulin resistance and contribute to inflammation. In rodent models of obesity, lower adiponectin levels increase the susceptibility to cognitive decline in experimental procedures [51], and adiponectin administration can improve cognitive impairment induced by insulin resistance [52]. Consequently, the reduction of adiponectin and the accompanying elevated levels of NSE in children with obesity raise the need to assess whether these children are at increased risk for developing cognitive impairment.

In contrast to adiponectin, resistin is an adipokine associated with neuroinflammation, obesity development, and cardiovascular risk [53–56]. We found higher levels of resistin in children with obesity. Data from mouse models show that adiponectin, but not resistin, can prevent the decrease in the cerebral uptake of glucose and cognitive decline in the brain induced by high-fat diet-induced obesity. In obese mice, resistin worsened the glucose control, dyslipidemia status, and cognitive performance [57].

Of note, mean serum NSE levels in control subjects without neuropathological conditions have been reported to be approximately 10 ng/mL with no age or gender effects [58, 59]. In adults, a study found that lower gray matter density, as measured by magnetic resonance imaging (MRI), correlated with circulating NSE levels in subjects with overweight and obesity (aged 20–41), even when NSE levels were in the non-pathological range [8]. These results suggest that NSE levels might indicate other disturbances beyond neuronal death since the level of NSE reported in adults with obesity (6.8 ng/mL) [19] is relatively lower than those reported in traumatic brain injury (191.38 ng/L) [60]. Moreover, S100β and NSE levels were reduced as weight and fat mass percentage in subjects with obesity after exercise training, suggesting S100β and NSE could be relevant indicators of health outcomes [19].

Our study has some limitations, such as being cross-sectional, having a limited number of samples, and lacking neuroimaging techniques to explore the relationship between the circulating levels of S100β and NSE and cognitive performance. Nonetheless, this is the first study showing that Mexican children with obesity have higher serum concentrations of S100β and NSE, and that these molecules are correlated with adipokines that play a critical role in the development of neuroinflammation and potentially cognitive impairment.

## Conclusion

Childhood obesity is a rising problem in modern society. Furthermore, Mexican children are at higher risk of developing type 2 diabetes and cardiovascular diseases according to their ethnicity [61, 62]. Our results provide preliminary evidence to support that obesity during childhood might increase the susceptibility to neuronal impairment. Further studies are needed in other ethnical populations, as well as in preschoolers and adolescents. Moreover, longitudinal studies that include interventions that impact the S100β and NSE levels, such as diet, exercise, and sleep, are critical in understanding the mechanisms in body-brain communication reflected in changes of circulating S100β and NSE.

## Supplementary information

Correlation of NSE and S100b

## Data Availability

The data supporting this study’s findings are available from the corresponding author upon reasonable request.
