# Supplementary material for: Serum S100β and neuron-specific enolase correlate with obesity parameters in Mexican children
Source: Int J Obes (Lond). 2025 Nov 24;50(2):378–85. doi: 10.1038/s41366-025-01942-y (PMC12913025; doi:10.1038/s41366-025-01942-y)
Supplement: Supplementary file 1 — Correlation of NSE and S100b [file 41366_2025_1942_MOESM1_ESM.pdf]

Correlation of NSE

	NSE vs. S100b	NSE vs. BDNF	NSE vs. Leptin	NSE vs. Adiponectin	NSE vs. Resistin	NSE vs. Insulin	NSE vs. BW	NSE vs. Age	NSE vs. BMI percentil	NSE vs. H-W index	NSE vs. Glucose	NSE vs. Cholesterol	NSE vs. TG	NSE vs. HDL Ch	NSE vs. LDL Ch	NSE vs. HOMA IR
**Pearson r**																
**r**	0.1676	-0.1676	-0.2028	-0.4972	0.2575	0.1143	0.2568	-0.1471	0.5294	0.4863	0.1361	-0.09978	0.1131	-0.2136	-0.02689	0.1017
**95% confidence interval**	-0.1023 to 0.4144	-0.3774 to 0.05854	-0.4122 to 0.02698	-0.6489 to -0.3074	0.03553 to 0.4552	-0.1125 to 0.3298	0.03484 to 0.4546	-0.3592 to 0.07952	0.3466 to 0.6735	0.2944 to 0.6405	-0.09065 to 0.3494	-0.3167 to 0.1270	-0.1138 to 0.3288	-0.4176 to 0.01086	-0.2494 to 0.1983	-0.1251 to 0.3184
**R squared**	0.02808	0.02810	0.04112	0.2472	0.06628	0.01307	0.06595	0.02163	0.2803	0.2365	0.01852	0.009956	0.01279	0.04564	0.0007228	0.01034
**P value**																
**P (two-tailed)**	0.2214	0.1450	0.0832	<0.0001	0.0238	0.3222	0.0242	0.2018	<0.0001	<0.0001	0.2379	0.3879	0.3274	0.0621	0.8165	0.3788
**P value summary**	ns	ns	ns	****	*	ns	*	ns	****	****	ns	ns	ns	ns	ns	ns
**Significant? (alpha = 0.05)**	No	No	No	Yes	Yes	No	Yes	No	Yes	Yes	No	No	No	No	No	No
**Number of XY Pairs**	55	77	74	77	77	77	77	77	77	77	77	77	77	77	77	77

Correlation of S100

	S100b vs. NSE	S100b vs. BDNF	S100b vs. Leptin	S100b vs. Adiponectin	S100b vs. Resistin	S100b vs. Insulin	S100b vs. BW	S100b vs. Age	S100b vs. BMI percentile	S100b vs. H-W index	S100b vs. Glucose	S100b vs. Cholesterol	S100b vs. TG	S100b vs. HDL Ch	S100b vs. LDL Ch	S100b vs. HOMA IR
**Pearson r**																
**r**	0.1676	-0.06310	-0.1744	-0.3227	0.2041	-0.02629	0.003216	-0.2393	0.2990	0.2996	-0.07309	-0.1450	0.04126	-0.2759	-0.05676	-0.01908
**95% confidence interval**	-0.1023 to 0.4144	-0.3207 to 0.2032	-0.4203 to 0.09526	-0.5398 to -0.06529	-0.06209 to 0.4433	-0.2872 to 0.2383	-0.2599 to 0.2659	-0.4724 to 0.02523	0.03914 to 0.5209	0.03985 to 0.5214	-0.3297 to 0.1935	-0.3929 to 0.1226	-0.2241 to 0.3009	-0.5024 to -0.01407	-0.3150 to 0.2093	-0.2806 to 0.2451
**R squared**	0.02808	0.003981	0.03043	0.1041	0.04167	0.0006910	1.034E-05	0.05724	0.08938	0.08977	0.005342	0.02101	0.001702	0.07615	0.003222	0.0003640
**P value**																
**P (two-tailed)**	0.2214	0.6441	0.2027	0.0153	0.1313	0.8475	0.9812	0.0757	0.0252	0.0249	0.5924	0.2864	0.7627	0.0395	0.6778	0.8890
**P value summary**	ns	ns	ns	*	ns	ns	ns	ns	*	*	ns	ns	ns	*	ns	ns
**Significant? (alpha = 0.05)**	No	No	No	Yes	No	No	No	No	Yes	Yes	No	No	No	Yes	No	No
**Number of XY Pairs**	55	56	55	56	56	56	56	56	56	56	56	56	56	56	56	56
